# The role of CXCL10 in prognosis of patients with colon cancer and tumor microenvironment remodeling

**DOI:** 10.1097/MD.0000000000027224

**Published:** 2021-09-24

**Authors:** Weiwei Song, Hongli Yin, Chenguang Han, Qiantai Mao, Jing Tang, Zhaoshuai Ji, Xu Yan, Lan Wang, Shengnan Liu, Chao Ai

**Affiliations:** a Beijing Tsinghua Changgung Hospital, School of Clinical Medicine, Tsinghua University. Beijing, China; b German Research Center for Environmental Health, 85764, Neuherberg, Germany; c College of Nankai University, Tianjin, China.

**Keywords:** colon adenocarcinoma, colorectal cancer, tumor microenvironment

## Abstract

**Backgroung:**

Tumor microenvironment (TME) has gradually emerged as an important research topic in the fight against cancer. The immune system is a major contributing factor in TME, and investigations have revealed that tumors are partially infiltrated with numerous immune cell subsets.

**Method:**

We obtained transcriptome RNA-seq data from the the Cancer Genome Atlas databases for 521 patients with colon adenocarcinoma (COAD). ESTIMATE algorithms are then used to estimate the fraction of stromal and immune cells in COAD samples.

**Result:**

A total of 1109 stromal-immune score-related differentially expressed genes were identified and used to generate a high-confidence protein–protein interaction network and univariate COX regression analysis. C-X-C motif chemokine 10 (CXCL10) was identified as the core gene by intersection analysis of data from protein–protein interaction network and univariate COX regression analysis. Then, for CXCL10, we performed gene set enrichment analysis, survival analysis and clinical analysis, and we used CIBERSORT algorithms to estimate the proportion of tumor-infiltrating immune cells in COAD samples.

**Conclusion:**

We discovered that CXCL10 levels could be effective for predicting the prognosis of COAD patients as well as a clue that the status of TME is transitioning from immunological to metabolic activity, which provided additional information for COAD therapies.

## 1 Introduction

Colorectal cancer (CRC) is one of the most frequent malignant gastrointestinal tumors in the world, with a significant morbidity and mortality rate. The most common form is colon adenocarcinoma (COAD).^[1]^ In recent years, greater progress has been made in the field of CRC research. A molecular cluster known as miR-371 to 373, which is primarily responsible for regulating cancer metastasis in CRC patients, has been discovered, which could help researchers in developing new therapies that effectively inhibit tumor growth.^[2]^ Fusobacterium nucleatum, a gram-negative oral anaerobe, has been shown to accelerate CRC by Rubinstein et al.^[3]^ This study could help researchers identify and treat malignant CRC more easily, as well as explain why some patients’ disease progresses much faster than others, which could be due to the presence of this bacteria in the mouth.^[3]^ Kumaradevan et al^[4]^ discovered a new target protein, c-Cbl, that may improve CRC patient survival. The signaling pathway that drives tumor progression is a promising target for systemic therapy in CRC. In addition to the WNT and MAPK pathways, the NOTCH signaling pathway has been found to be activated in most tumors. According to Schmidt et al,^[5]^ high NOTCH activity indicates a subpopulation of colon cancer cells with low levels of WNT and MAPK signals and a significant epithelial phenotype.^[5]^ Drugs targeting the MAPK signaling pathway have a limited inhibitory function on tumor growth, since it will cause the expansion of NOTCH highly activated cancer cells, but if the NOTCH signaling pathway is targeted, cancer cells with high MAPK activity will increase. They discovered that tumor cell subpopulations are highly adaptable, which is also a mechanism that leads to tumor drug resistance. In vivo, combined therapy targeting NOTCH and MAPK signaling can have a significant tumor suppressive effect. Now, COAD is primarily treated surgically, with additional comprehensive treatments such as chemotherapy, radiotherapy, or targeted therapy. The survival rate of colon cancer has improved with the development of surgical techniques, but metastasis and recurrence of the tumor still resulted in a poor prognosis for patients. As a result, research into the carcinogenesis and therapeutics of COAD is required.

Tumor microenvironment (TME) has increasingly become an important study area to overcome cancer in recent years. The internal environment in which tumor cells are formed and reside is known as TME. Tumors are not merely made up of malignant cells, and they are also not unaffected by their surroundings. They could recruit and corrupt nonmalignant cells including fibroblasts and immune cells linked to cancer, as well as cytokines and chemokines, to create TME. Tumor growth is influenced by changes in the TME, such as stroma, blood vessels, and infiltrating inflammatory cells, as well as genetic changes in malignant tumor cells. TME has long been a hot area in tumor research, with significant implications for our understanding of tumor occurrence, progression, and metastasis. It is also useful in the diagnosis, prevention, and prognosis of cancers. TME is largely caused by the immune system, and investigations have revealed that tumors are partially infiltrated with numerous immune cell subsets. These immune cells and matrix components recruited and activated by tumor cells generate a tumor-inhibiting inflammatory milieu that inhibits tumorigenesis and development during tumor colonization or early stages of tumor growth. The relevant effector cells in the TME, on the other hand, are exhausted or remodeling after continuous tumor antigen stimulation and immune activation and are unable to perform normal functions or even promote the malignant characterization of tumors, resulting in an immunosuppressive microenvironment. Because tumor types differ, so do infiltrating immune cells, and even among patients with the same pathological type of tumor, infiltrating immune cell subsets differ. As a result, establishing an effective immune evaluation system and taking advantage of TME-targeted immunotherapy strategies to stimulate or restore the immune system's inherent tumor suppressive ability and reshape the active immune microenvironment has become a pressing question.

In this paper, we used Estimate algorithms to calculate the fraction of stromal and immune cells in COAD samples from The Cancer Genome Atlas (TCGA) database, and CIBERSORT algorithms to estimate the proportion of tumor-infiltrating immune cells (TICs) in COAD samples. Finally, the C-X-C motif chemokine 10 (CXCL10) predictive marker has been identified. CXCL10 is a member of the CXC chemokine family, and it binds to the CXCR3 receptor to mediate immune responses on activated leukocytes.^[6]^ CXCL10 gene is primarily expressed by fibroblasts, endothelial cells, hepatocytes, keratinocytes, and other cells. It has a low expression in a steady-state cell, but it is up-regulated in response to inflammatory cytokines. CXCL10 gene expression is primarily induced by IFN-γ, and its primary function is to chemoattract T lymphocytes, monocytes, and natural killer cells to the site of inflammation, where they can exert anti-inflammatory and immune effects. The CXCL10 gene is also a vascular inhibitory factor that can attract antitumor T lymphocytes and have antitumor effects, but tumor cells’ autocrine CXCL10 signal can enhance tumor cell proliferation, angiogenesis, and metastasis.^[7]^ It has an essential role in kinds of human diseases, such as tumor development, metastasis and dissemination infectious diseases, chronic inflammation, immune dysfuntion, etc. In addition, it has been identified as a major biological marker in various disease, such as chronic graft-versus-host disease,^[8]^ Kawasaki disease,^[9]^ and heart failure and left ventricular dysfunction,^[10]^ etc. Endogenous CXCL10 is critical for cancer cell recruitment to bone, can promote osteoclast differentiation, and is a key factor in the formation of osteolytic bone metastasis.^[11]^ We started with differentially expressed genes (DEGs) generated by comparing immune components and stromal components in COAD samples and discovered that the CXCL10 could be a potential indicator of TME status change in COAD.

## 2 Methods

### 2.1 Data download

Transcriptome RNA-seq datasets and clinical data of 521 COAD cases (normal samples, 41 cases; tumor samples, 480 cases) were downloaded from TCGA database (https://portal.gdc.cancer.gov/).

### 2.2 Survival analysis

The survival analysis was performed using R and the packages survival and survminer. The survival curve was plotted using the Kaplan–Meier method, with log rank as the statistical significance test. A difference of *P* < .05 indicated statistical significance.

### 2.3 Identification of DEGs

According to the results of the ESTIMATE algorithms, all COAD cases were divided into high and low groups based on immune and stromal scores depending on the comparison to the median score. The package limma was used to perform gene expression differentiation analysis, and DEGs were created by comparing high-score samples to low-score samples, with a threshold value of false discovery rate <0.05 and fold change (FC) >1 by a Wilcoxon rank-sum test.

### 2.4 Gene ontology (GO) and Kyoto Encyclopedia of Genes and Genomes (KEGG) pathway enrichment analysis

The “clusterProfiler,” “ggplot2,” “org.Hs.eg.db,” and “enrichplot” packages were used to select the 521 DEGs for gene ontology (GO) and Kyoto Encyclopedia of Genes and Genomes (KEGG) pathway enrichment analyses. The samples with *P* values and q values less than .05 were considered significantly enriched.

### 2.5 PPI network

The protein–protein interaction (PPI) network was built using the search tool for the retrieval of interacting gene database. To construct the network, nodes with a confidence score of interactive relationship >0.95 was set as the cutoff criterion using Cytoscape software.

### 2.6 COX regression analysis and heatmaps

For univariate COX regression, the R language was utilized, along with the package survival. The plot depicted the top 30 genes in univariate COX, arranged by *P* value from small to large.

Heatmaps of DEGs were created using the R language and the pheatmap package.

### 2.7 Gene set enrichment analysis (GSEA)

The target sets for gene set enrichment analysis (GSEA) were the Hallmark and C7 gene sets v6.2 collections from the Molecular Signatures Database, which were used with the software gsea-3.0 from the Broad Institute. GSEA was performed on the entire transcriptome of all tumor samples. Only gene sets with NOM *P* value < .05 were considered as statistically significant.

### 2.8 Evaluation of tumor infiltrated immune cells (TICs)

The CIBERSORT algorithm was used to calculate the fractions of tumor-infiltrated immune cells in all tumor cases. When the CIBERSORT *P* value was <.05, the data were filtered and selected for further analysis using the R language's “limma” package. The R packages “limma,” “vioplot,” “gglot2,” “ggpubr,” and “ggExtra” were used for difference and correlation analyses. Using the Wilcoxon rank-sum and Pearson coefficient tests, *P* values <.05 were considered statistically significant. The Tumor Immune Estimation Resource 2.0 database (http://timer.cistrome.org/) was also used for the cumulative survival analysis. n this study, the proportion and composition of 22 kinds of TICs in tumor were analysed.

### 2.9 Ethical statement

This article does not contain any studies involving human participants performed by any of the authors. The authors are responsible for all parts of the work, including ensuring that any concerns about the accuracy or integrity of any portion of the work are thoroughly examined and resolved. The present study was authorized by the Research Ethics Review Board of Beijing Tsinghua Changgung Hospital.

## 3 Results

### 3.1 The correlation of scores with pathology and prognosis in COAD patients

**Figure 1:**
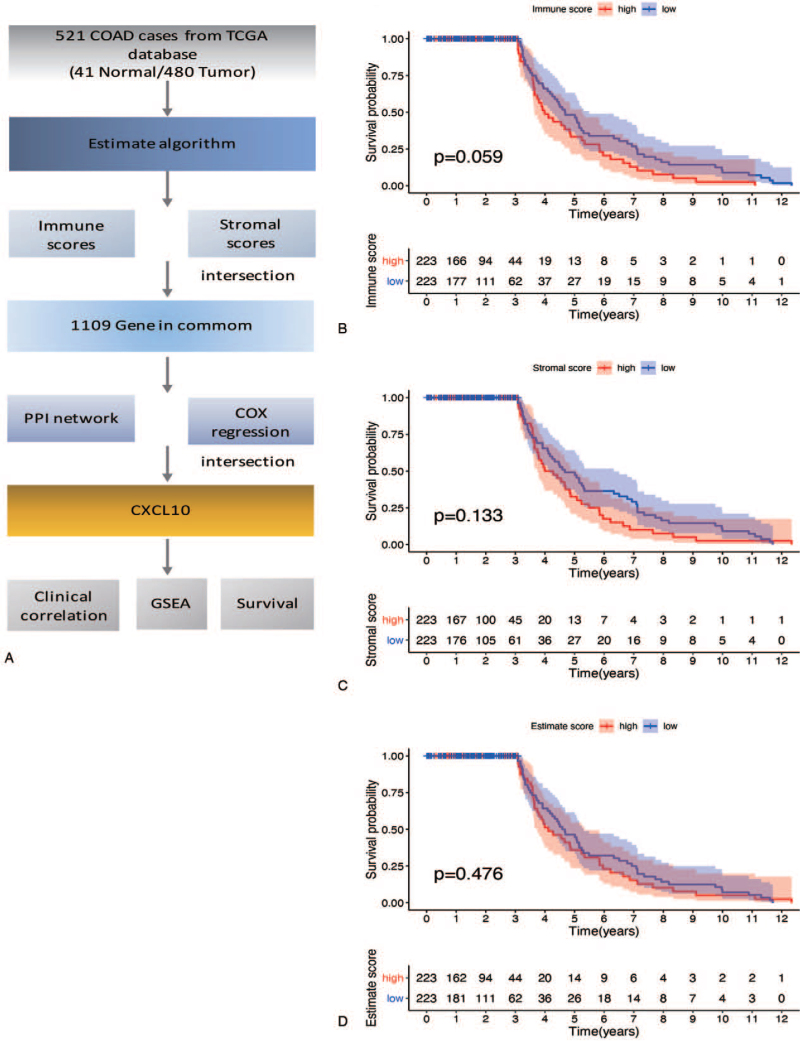
(A) Workflow of this study. (B–D) Kaplan–Meier survival analysis for COAD patients divided into high or low score determined by the comparison with the median. COAD = colon adenocarcinoma, CXCL10 = C-X-C motif chemokine 10, GSEA = gene set enrichment analysis, PPI = protein–protein interaction, TCGA = the Cancer Genome Atlas.

We obtained transcriptome RNA-seq data from TCGA databases for 521 COAD patients. The clinicopathological characteristics of these COAD patients are depicted in Figure 1 (Table 1). The fraction of stromal and immune cells in COAD samples was then estimated using ESTIMATE algorithms.^[12]^ A total of 1109 stromal-immune score-related DEGs were identified and used to generate a high-confidence PPI network and univariate COX regression analysis. CXCL10 was identified as the core gene by intersection analysis of data from the PPI network and univariate COX regression analysis. Then, for CXCL10, we performed GSEA, survival analysis, and clinical analysis (Fig. 1A).

**Table 1:** The clinicopathological characteristics of COAD patients.

Clinical characteristics	Type	Total (452)	%
Age	≤65	185	40.90
	>65	267	59
Gender	Male	238	52.60
	Female	214	47.30
Stage	Stage I	77	17
	Stage II	178	39.40
	Stage III	125	27.70
	Stage IV	62	13.70
T classification	T1	10	2.20
	T2	77	17
	T3	281	62
	T4	83	18.40
M classification	M0	334	74
	M1	62	13.70
N classification	N0	269	59.50
	N1	103	22.80
	N2	79	17.50

COAD = colon adenocarcinoma, M = metastasis, N = lymph node staging, T = tumor infiltration depth.

Kaplan–Meier survival analysis was used to determine the potential association between prognosis and elevated immune, stromal, and estimate scores. Although there is no statistically significant difference (*P* = .059), immune score showed a potential negative correlation with overall survival rate. The stromal score (*P* = .33) and estimate score (*P* = .476) were not related to overall survival rate (Fig. 1B–D).

**Figure 2:**
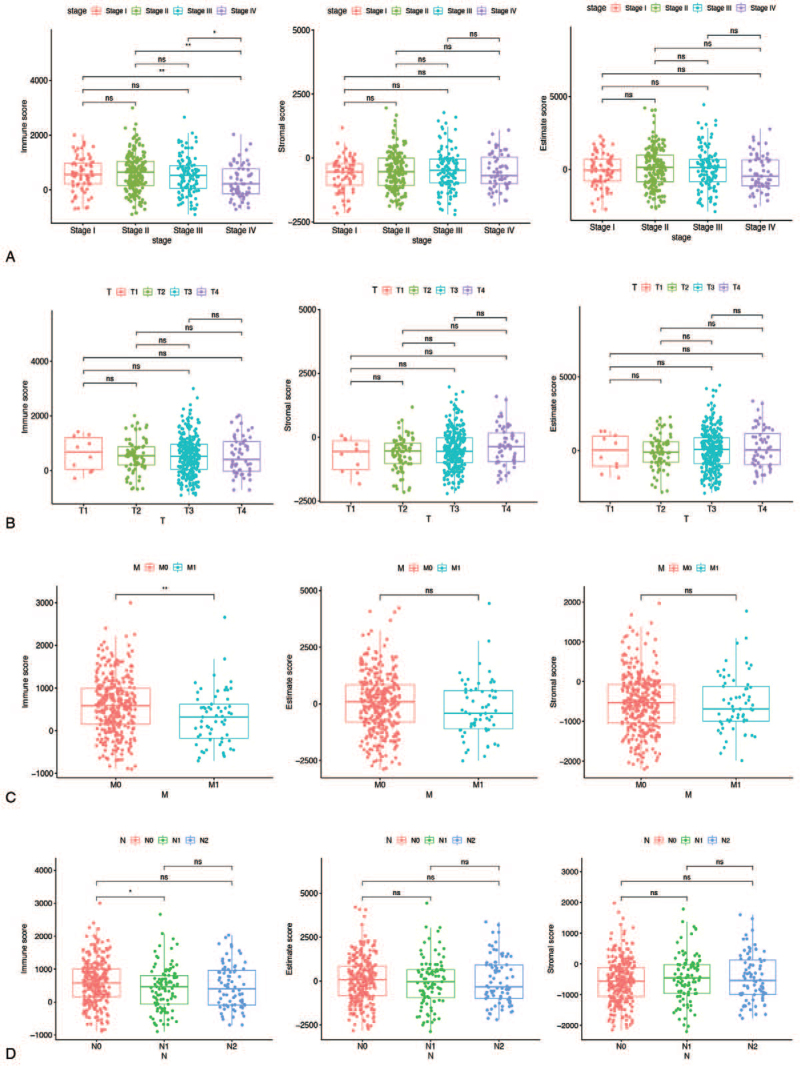
Association with immune score, stromal score, estimate score, and clinicopathologic characteristics. (A) Clinical stage (B) T classification (C) M classification (D) N classification. M = metastasis, N = lymph node staging, T = tumor infiltration depth.

The relationship between stromal and immune scores and COAD patient pathologic characteristics was investigated by calculating score distributions across tumor stages and histology classifications (TNM stages). As shown in Figure 2A, there was a significant negative correlation between immune scores and tumor stage, with immune scores decreasing in the late tumor stage. Furthermore, the immune score has a different distribution in the M and N of TNM stage classifications (Fig. 2C and D). The above findings indicated that the distribution of immune components was related to the progression of COAD.

### 3.2 DEGs of immune scores and stromal scores

**Figure 3:**
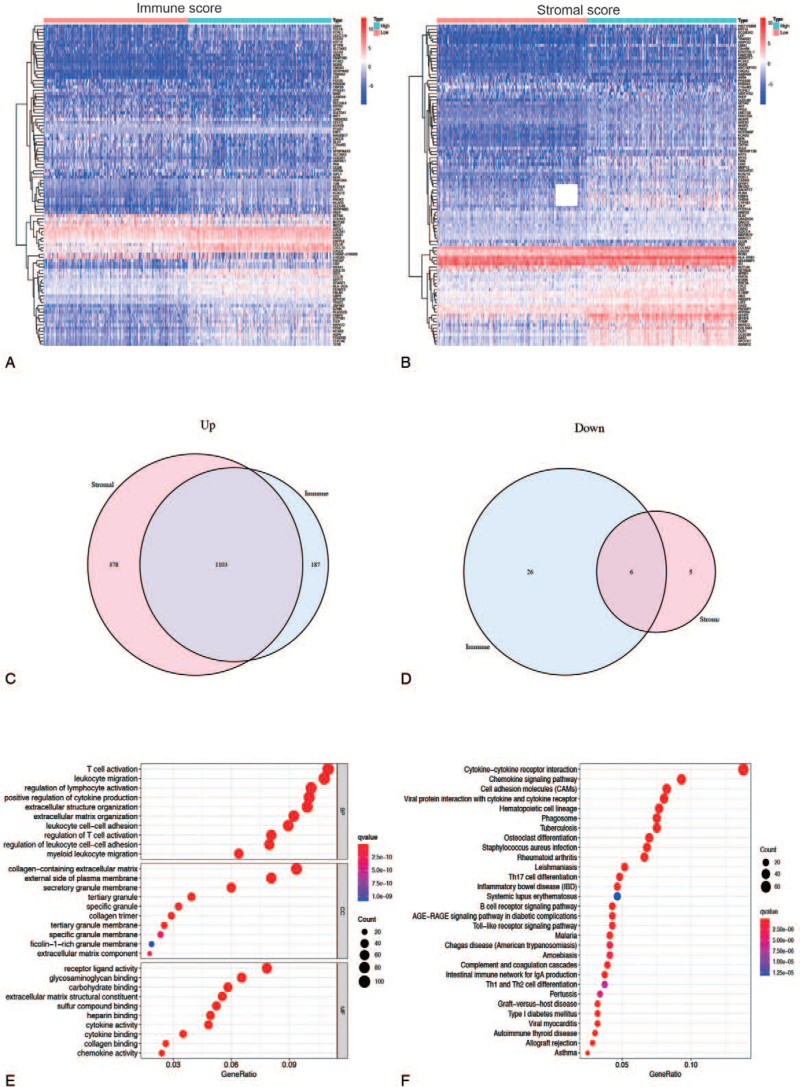
Identification of DEGs. (A–B) Heatmap for DEGs via comparing the high score samples to the low score samples in immune score and stromal score separately. DEGs were determined by Wilcoxon rank sum test with q = 0.05 and fold-change >1 as the statistically significant. (C, D) The Venn plots showed the up-regulated and down-regulated DEGs shared by immune score and stromal score, and q < 0.05 and fold-change >1 after log2 transformation as the DEGs significance filtering threshold. (E, F) GO functional and KEGG pathway enrichment analyses for 1109 DEGs in the object module. DEGs = differentially expressed genes, GO = gene ontology, KEGG = Kyoto Encyclopedia of Genes and Genomes.

To determine the relationship of gene expression profiles with immune and stromal scores, we analyzed gene microarray data from 521 COAD cases obtained from TCGA database. We obtained 1322 DEGs from the immune score and 1692 DEGs from the stromal score by comparing the high and low score groups. The heatmap displayed distinct gene expression profiles of the top 50 DEGs from low and high scoring immune or stromal groups (Fig. 3A and B). There were 1290 up-regulated genes and 32 down-regulated genes among the immune score (Fig. 3C). Similarly, 1681 genes were up-regulated, and 11 genes were down-regulated based on stromal score (Fig. 3D). The intersection analysis of Venn diagrams revealed 1103 co-upregulation genes in groups with high immune and stromal scores, and 6 co-downregulation genes in groups with low immune and stromal scores. The 1109 DEGs were then subjected to GO enrichment analysis to determine their potential function. The enriched GO terms discovered primarily include T-cell activation, leukocyte migration, and lymphocyte activation regulation, all of which are immune-related GO terms (Fig. 3E). Furthermore, the KEGG enrichment pathways are primarily enriched in chemokine signaling pathways, cytokine–cytokine receptor interaction, and chemokine signaling pathways, as well as cell adhesion molecules related to immune response (Fig. 3F).

### 3.3 Intersection analysis of protein–protein interaction network (PPI) and univariate COX

**Figure 4:**
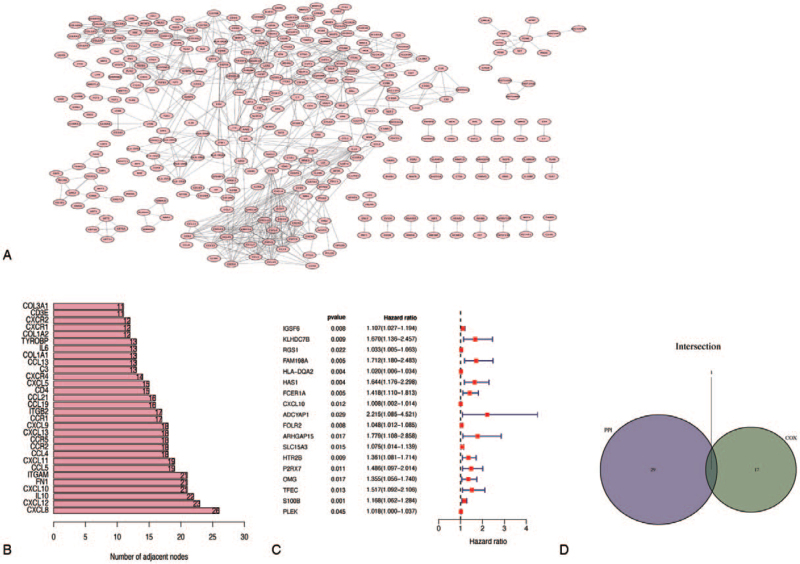
Intersection analysis of PPI and univariate COX. (A) PPI interaction network of DEGs, and the interaction confidence value setting is greater than 0.95. (B) The top 30 genes sorted by the number of nodes. (C) Univariate COX regression analysis with *P* < .05 setting. (D) Interaction analysis of PPI and univariate COX. DEGs = differentially expressed genes, PPI = protein–protein interaction.

We screened 1109 DEGs into a PPI network using the search tool for the retrieval of interacting gene database and Cytoscape software (Fig. 4A), and the top 30 genes ranked by number of nodes are shown (Fig. 4B).

To further investigate the significant elements of these 1109 DEGs in the survival prognosis of COAD patients, a univariate Cox regression analysis was performed, and the top 18 genes sorted by p value were identified (Fig. 4C). The top 50 nodes in the PPI network were then intersected with the top 18 significant factors in univariate COX regression. According to the results of the above analysis, only 1 overlapping gene, CXCL10, can be identified (Fig. 4D).

### 3.4 The relationship between CXCL10 expression and survival and TNM stage classification in COAD Patients

**Figure 5:**
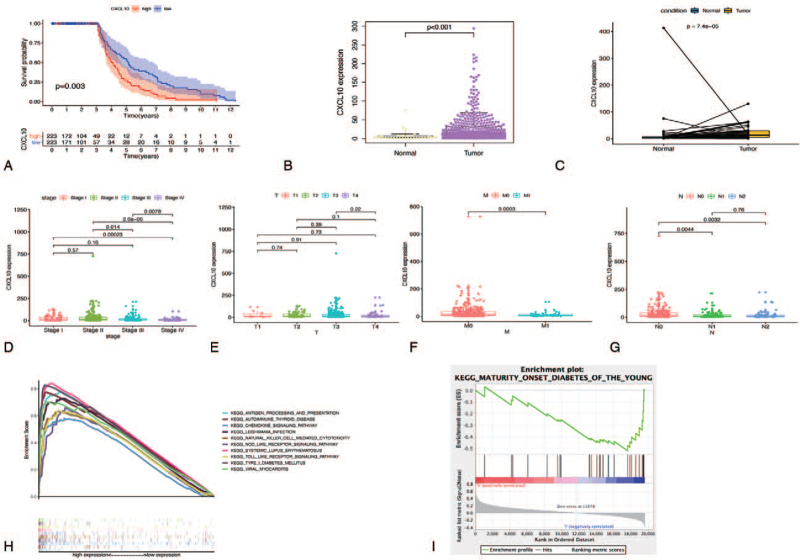
Analysis of CXCL10. (A) Kaplan–Meier survival analysis for COAD patients with high or low CXCL10 expression, CXCL10 cutoff/median expression is 11.0901099. (B) CXCL10 expression in tumor vs normal sample. CXCL10 cutoff/median expression is 11.0901099. (C) CXCL10 expression in the normal and tumor sample of the same patient. (D–G) Association with CXCL10 and clinicopathologic characteristics. (D) Clinical stage. (E) T classification. (F) M classification. (G) N classification. (H, I) GSEA analysis for samples with different CXCL10 expression. COAD = colon adenocarcinoma, CXCL10 = C-X-C motif chemokine 10, GSEA = gene set enrichment analysis, KEGG = Kyoto Encyclopedia of Genes and Genomes, M = metastasis, N = lymph node staging, T = tumor infiltration depth.

CXCL10 is secreted by leukocytes and tissue cells in response to IFN-γ,^[13,14]^ which is abundant in a variety of human diseases including infectious diseases, immune dysfunction, inflammatory diseases, and cancer.^[13–16]^. To investigate the possible link between CXCL10 expression and overall survival of COAD samples, All COAD samples were divided into 2 groups based on CXCL10 median expression: CXCL10 high-expression and CXCL10 low-expression. Table 2 shows the detailed clinical characteristics of COAD patients in the CXCL10 high or low expression groups. The survival curve revealed that COAD patients with high CXCL10 expression had a lower survival rate than those with low expression (Fig. 5A). And, as shown in Figure 5B, CXCL10 expression was significantly higher in tumor samples than in normal samples. Because the number of tumor samples differed from the number of normal samples, we used matched paired analysis to determine the expression of CXCL10 in normal and tumor tissues from the same patient. The pairing analysis of normal and tumor tissues derived from the same patient yielded similar results (Fig. 5C). There were significant differences in CXCL10 expression between the tumor stages IV and I, ranging from low to high. However there is no difference between stage I and II, as well as stage I and III (Fig. 5D). There was no significant difference in CXCL10 expression between the 4 stages in terms of tumor infiltration depth (T) (Fig. 5E). In terms of metastasis (M), the relationships between CXCL10 expression in the 2 stages were as follows: M0 > M1 (with statistical significance) (Fig. 5F). Finally, in terms of lymph node staging (N), CXCL10 expression follows the pattern: N0 > N1, N0 > N2, with statistical significance, but no difference between N1 and N2 (Fig. 5G). These findings suggested that CXCL10 expression in TME was associated with a poor prognosis in COAD patients. Moreover, CXCL10 was not significantly related to T stage (*P* > .05), but it was related to tumor stages, N stage and M stage (*P* < .05). GSEA of TCGA was performed to retrieve biological processes enriched in CXCL10 with highly and lowly expressed samples to analyze the potential mechanisms of CXCL10 in association with COAD. According to Figure 5H, the genes in the CXCL10 high-expression group were primarily enriched in “antigen processing and presentation,” “autoimmune thyroid disease,” “chemokine signaling pathway,” “leishmania infection,” and “systemic lupus erythematosus.” The genes were enriched in “maturity onset diabetes of the young” for the CXCL10 low-expression group (Fig. 5I).

**Table 2:** The COAD patient clinicopathological characteristics for both CXCL10 high expression and low expression groups.

Clinical characteristics	Type	
CXCL10 expression	Low 216 (52%)	High 236 (47.8%)
Gender		
Male	2 (0.93%)	236 (100%)
Female	214 (99%)	0
Stage		
Stage I	76 (35%)	0
Stage II	140 (65%)	37 (16.7%)
Stage III	0	124 (55.9%)
Stage IV	0	61 (27.4%)
T classification		
T1	10 (4.6%)	0
T2	77 (35.6%)	0
T3	129 (59.7%)	181 (77%)
T4	0	54 (23%)
M classification		
M0	216 (100%)	118 (65.5%)
M1	0	62 (34.4%)
N classification		
N0	216 (100%)	53 (22.5%)
N1	0	103 (43.6%)
N2	0	80 (33.9%)

COAD = colon adenocarcinoma, CXCL10 = C-X-C motif chemokine 10, M = metastasis, N = lymph node staging, T = tumor infiltration depth.

### 3.5 The relevance of CXCL10 with the proportion of TICs

**Figure 6:**
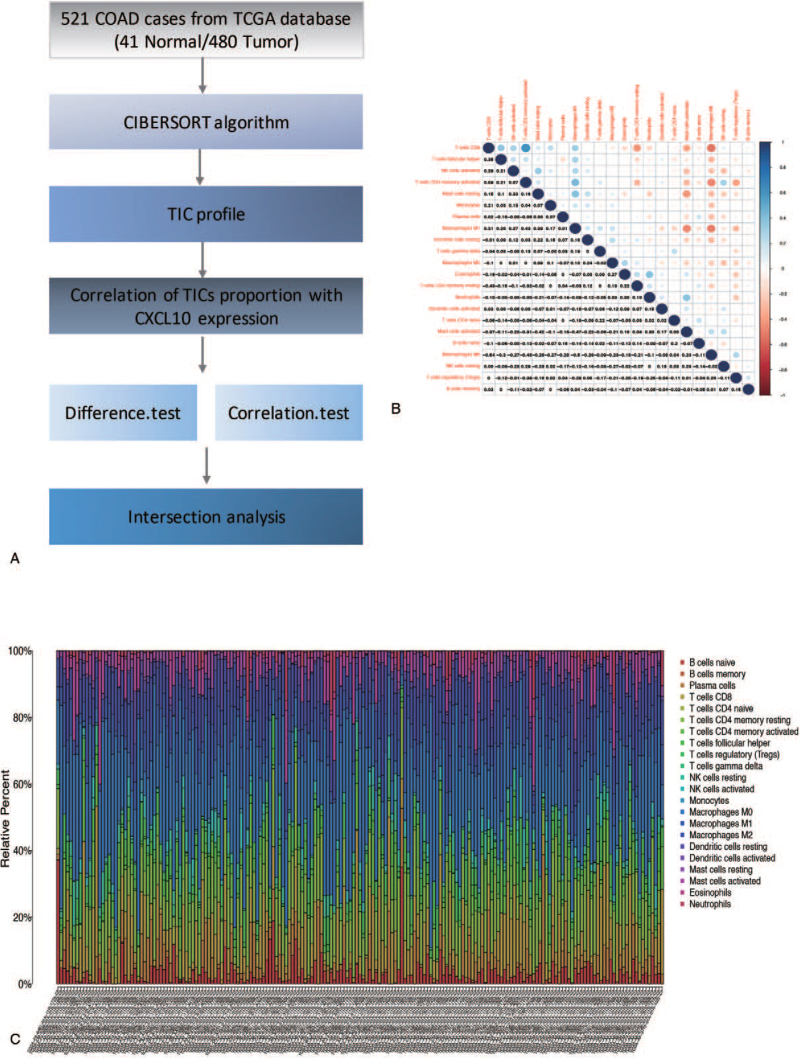
The distribution of immune infiltration in COAD. (A) The workflow of this part analysis. (B) The proportions of different immune cells in each COAD sample. (C) Correlation of all type of immune cells. Blue plot indicated negatively related immune cells, red plot indicated positive related immune cells. COAD = colon adenocarcinoma, CXCL10 = C-X-C motif chemokine 10, TCGA = the Cancer Genome Atlas, TICs = tumor-infiltrating immune cells.

**Figure 7:**
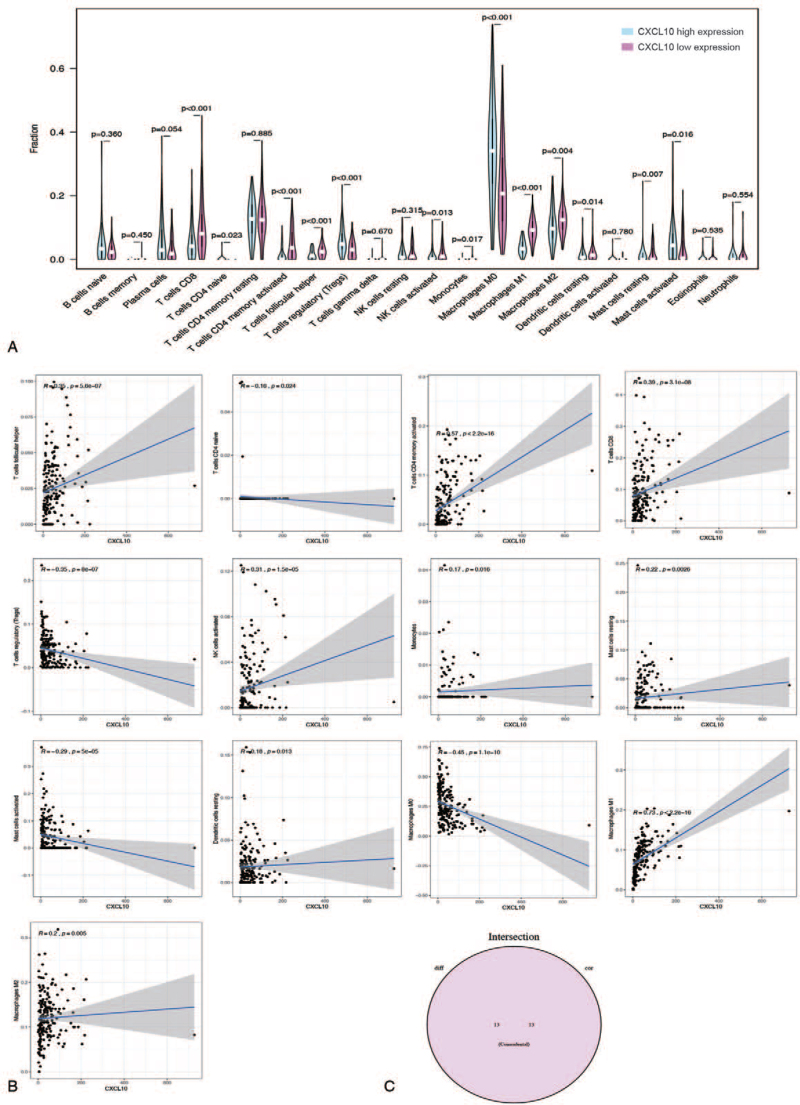
The correlation of TICs proportion with CXCL10 expression. (A) High or low expression of CXCL10 in different immune cells. (B) The correlation of TICs proportion with the CXCL10 expression, *P* < .05 as statistically significant. (C) Intersection analysis. CXCL10 = C-X-C motif chemokine 10, TICs = tumor-infiltrating immune cells.

The CIBERSORT algorithm was used to analyze 521 cases from the TCGA dataset to estimate the relative proportion of TICs in COAD samples^[17]^ (Fig. 6A). Then, a correlation analysis between CXCL10 and TICs was carried out (Fig. 6B). Figure 7A depicts the proportion of 22 types of TICs in 521 cases of COAD, and the proportion of TICs varies by sample. Furthermore, as shown in Figure 6C, there is a strong correlation between the different 22 TICs in the COAD microenvironment. For example, macrophage M0 is most positively correlated with T cell CD8, indicating that these 2 types of immune cells work together to help COAD patients. Furthermore, T cells CD8 memory activation is negatively correlated with T cells CD4 memory activation, indicating that these 2 types of immune cells have an antagonistic effect in COAD patients. The violin plot in Figure 7A demonstrated the ratio differentiation of 22 TICs between COAD tumor samples with low or high CXCL10 expression. Afterward, we conducted a correlation analysis between CXCL10 expression and the proportion of TICs (Fig. 7B). Finally, the intersection analysis of difference and correlation analyses revealed that 13 different types of TICs were associated with CXCL10 expression (Fig. 7C). There is a positive correlation between 9 types of TICs and CXCL10 expression, while the remaining are negatively correlated with CXCL10 expression. According to the data presented above, CXCL10 expression levels correlated with TME immune activity.

## 4 Discussion

CRC is the most common cancer worldwide, with a high morbidity and mortality rate. Every year, approximately 1.3 million new cases are diagnosed, and nearly 700,000 people die from this cancer. Precision medicine's advancement holds great promise for CRC patients.

However, the high rate of metastasis and invasiveness of CRC has been a bottleneck in reducing mortality, resulting in an unsatisfactory 5-year survival rate.^[18]^ As a result, understanding the biological mechanisms underlying cancer progression and metastasis is critical for clinical treatment of patients.

In this paper, we used the TCGA database to collect transcriptome RNA-seq data and clinical information from COAD patients. TME-related DEGs associated with survival and TNM stage classification in COAD patients are identified. Finally, through intersection analysis, CXCL10 was discovered, which could be a new marker for the status of TME in COAD patients. Cancer treatment failure and patient death are primarily caused by tumor metastasis. Its molecular mechanism is complicated, involving multiple steps, stages, and gene changes. TME is important in the process of tumor metastasis because it provides a place for tumor cells to survive. Immune cells and stromal cells are the 2 main types of nontumor components found in TME, and they have been proposed to be useful for tumor diagnosis and prognostic evaluation. We discovered that the proportion of immune and stromal components in TME are not related to patient prognosis but are related to COAD progression by analyzing the TGCA–COAD database. Tumor metastasis, for example, is inversely proportional to immune score. These findings highlight the importance of studying the interactions of different types of cells in TME and will aid in the development of new immunotherapy options. The immune score can more effectively and accurately predict the risk of CRC progression, according to a recent important paper report published in *The Lancet*.^[19]^ Immune cell infiltration in the tumor can be used as a good reference indicator to indicate the development of CRC, which can be used as a potential prognostic indicator. Based on traditional TNM staging, combined with an immune score, which can improve tumor prognosis assessment. Recurrence risk classification based on immune scores can be used to improve individualized treatment strategies, particularly in guiding chemotherapy adjustment. They demonstrated that the immune score, as an analysis of tumor immune responsiveness, can better guide individualized and accurate anticancer treatment, which will almost certainly change clinical research design ideas. It will eventually help to improve cancer prevention and control around the world. A differential analysis was performed on samples with high and low scores to determine the exact changes in gene expression profiles related to immune and stromal components in TME. A total of 1109 DEGs were obtained by analyzing the low-score shared genes and the high-score shared genes using intersection analysis, which could be used to predict TME status. According to the GO enrichment analysis, DEGs are associated with immune-related terms. According to KEGG pathway enrichment analysis, these DEGs are primarily enriched in the cytokine cytokine receptor signaling pathway, among others. As a result, DEG is linked to immune regulation, implying that the involvement of immune factors is the primary feature of TME in COAD. Then, we discovered that the level of CXCL10 expression has a significant relationship with patient prognosis and the progression of COAD. As a result of these findings, CXCL10 has the potential to be a prognostic indicator as well as a therapeutic target for TME in COAD.

Wennerberg et al^[22]^ discovered that CXCL10 can directly bind to the CXCR3 receptor on cancer cells and inhibit their proliferation. It can also interfere with the expression of oncogenic factors like E6 and E7, which causes increased expression of p53 and promotes cancer cell death.^[20]^ CXCL10 also has an antitumor effect by regulating immunity; it can recruit a variety of immune cells to kill tumor cells directly. Liu et al^[21]^ demonstrated that esophageal squamous cell carcinoma secreted CXCL10 protein, which recruited CD8+T lymphocytes into cancer tissues and caused tissue damage. Patients with this type of cancer now have a better overall survival rate. The defective expression of CXCL10 in tumors may contribute to the immune escape mechanism.^[22]^ There have been few clinical studies on the relationship between CXCL10 and tumor therapeutic effect. Tamoxifen-treated breast cancer patients with high CXCL10 expression outlive those with low CXCL10 expression, according to studies, and this can be used as a useful predictor of tamoxifen therapy.^[23]^ According to studies on rectal cancer, the positive expression rate of CXCL10 in neoadjuvant radiochemotherapy (pathological complete response) is 73.7%, which is significantly higher than the 38.6% in pathological incomplete remission (non-pathological complete response), indicating that high expression of CXCL10 has higher treatment sensitivity, and CXCL10 is related to the efficacy and prognosis of rectal cancer.^[24]^ CXCL10 primarily exerts biological effects by binding to the CXCR3 receptor. On the 1 hand, it can inhibit tumor development by suppressing immunity, damaging immune responses, promoting tumor angiogenesis, and inhibiting apoptosis. On the other hand, it can promote tumor development by suppressing immunity, damaging immune responses, promoting tumor angiogenesis, and inhibiting apoptosis.^[25–28]^ The mechanism of its role in the occurrence and progression of tumors needs to be investigated further.

In our study, we performed GSEA enrichment analysis on the association between CXCL0 expression and TME and discovered that CXCL10 high-expression groups were primarily enriched in immune-related signaling pathways, indicating that CXCL10 may participate in the regulation of immune signaling pathways in TME. Finding new specific therapeutic targets has become a hot topic in tumor treatment research in recent years. CXCL10's diverse properties are expected to make it a new strategy for targeted tumor treatment, as well as a clinical tumor prognosis indicator.

## 5 Conclusion

We conducted a comprehensive analysis of the TME in COAD and predicted a prognostic indicator for COAD, providing a novel insight for COAD therapeutics.

## Author contributions

**Data curation:** Chenguang Han.

**Formal analysis:** Qiantai Mao, Xu Yan.

**Software:** Chenguang Han, Zhaoshuai Ji, Lan Wang, Shengnan Liu.

**Supervision:** Chao Ai.

**Writing – original draft:** Weiwei Song, Hongli Yin, Jing Tang.

**Writing – review & editing:** Chao Ai.
